# Bi-directional regulation between inflammation and stem cells in the respiratory tract

**DOI:** 10.1242/jcs.263413

**Published:** 2024-11-07

**Authors:** Jinwook Choi, Jakub Chudziak, Joo-Hyeon Lee

**Affiliations:** ^1^ School of Life Sciences, Gwangju Institute of Science and Technology, Gwangju 61005, Republic of Korea; ^2^ Cambridge Stem Cell Institute, Jeffrey Cheah Biomedical Centre, University of Cambridge, Cambridge CB1 0AW, UK; ^3^ Department of Physiology, Development and Neuroscience, University of Cambridge, Cambridge CB2 3EL, UK; ^4^ Developmental Biology Program, Sloan Kettering Institute, Memorial Sloan Kettering Cancer Center, New York NY 10065, USA

**Keywords:** Immune cells, Epithelial stem cells, Inflammation, Tissue regeneration, Stem cell fate, Niche remodeling, Respiratory tract, Aging

## Abstract

Inflammation plays a crucial role in tissue injury, repair and disease, orchestrating a complex interplay of immune responses and cellular processes. Recent studies have uncovered the intricate connection between inflammation and stem cell dynamics, shedding light on the central role of stem cells in tissue regeneration. This Review highlights the significance of inflammation in shaping epithelial stem cell dynamics and its implications for tissue repair, regeneration and aging. We explore the multifaceted interactions between inflammation and stem cells, focusing on how inflammatory signals affect stem cell behavior and fate as well as the remodeling of their niche in the respiratory tract. We also discuss the concept of ‘inflammatory memory’ in epithelial stem cells, where prior inflammatory stimuli endow these cells with enhanced regenerative potential and confer long-lasting protective mechanisms for maintaining tissue integrity and function. Furthermore, we review the impact of cell senescence induced by inflammation on tissue regeneration and aging, delving into the molecular mechanisms underlying the modulation of signaling pathways, epigenetic modifications and cellular crosstalk. Understanding these dynamic processes not only deepens our knowledge of tissue homeostasis and repair but also holds profound implications for regenerative medicine strategies aimed at preventing pulmonary diseases.

## Teaser

**Summary:** This Review highlights recent updates on how inflammation coordinates tissue homeostasis, regeneration and aging in epithelial tissues like the respiratory tract by regulating stem cells and their niches.

## Introduction

When a tissue is injured, it relies on a combination of regenerative and wound repair processes to maintain its integrity and function. Tissue-resident stem cells are central to these intertwined mechanisms. These cells are characterized by their ability to self-renew and differentiate, generating functional progeny to replace damaged or lost cells. Given their pivotal role in tissue regeneration, understanding how these stem cells perceive and respond to environmental changes, as well as orchestrate the tissue regeneration process, has become a key area of research. The behavior and fate decisions of stem cells are governed by intrinsic genetic and epigenetic regulatory programs integrated with specialized tissue microenvironments termed ‘niches’. Stem cell niches, first proposed by Raymond Schofield in 1978, represent specific locations that provide supportive signals that regulate stem cell behavior, restrict quiescence and promote differentiation (Schofield, 1978). Uncovering the precise cellular and molecular interactions between stem cells and other components of their respective niches is crucial to understanding how tissues maintain homeostasis and repair themselves in response to damage.

In recent years, immune cells have emerged as crucial components of the stem cell niche, playing a major role in both the maintenance of tissue homeostasis and regeneration. Tissue injury triggers an inflammatory cascade that coordinates immune responses at the wound site (Box 1). The importance of inflammation in this setting extends beyond its traditional functions, such as pathogen clearance, and is now recognized as a key part of the tissue regeneration process. Recent studies have underscored the essential role of regenerative inflammation, particularly that elicited by innate immune cells, such as macrophages, in reprogramming stem cells and remodeling their microenvironments (Arnold et al., 2007; Saclier et al., 2013; Su et al., 2015). However, if inflammation persists and becomes chronic, it hinders the repair process, leading to irreversible scarring and fibrosis. This shift from regenerative to pathological inflammation is implicated in various human diseases, including cardiovascular, neurodegenerative and metabolic pathologies, as well as aging-related conditions recently termed ‘inflammaging’ (Alfaddagh et al., 2020; Lee & Olefsky, 2021; Zhang et al., 2023). Understanding how these opposing types of inflammation affect stem cell behavior, and how stem cells influence local inflammatory responses, is crucial for better comprehending tissue regeneration and the early stages of pathological dysfunction that lead to disease.

**Box 1. The wound inflammatory response**

The inflammatory response is a key part of wound healing. Innate immune cells use the pattern recognition receptors (PRRs) found on their surface to detect infections through sensing of pathogen-associated molecular patterns (PAMPs), as well as cellular damage in the local environment, through sensing of damage associated molecular patterns (DAMPs) (Li and Wu, 2021). Inflammatory cells are recruited to the wound site via inflammatory cytokines, such as IL-1 and TNF (Kolaczkowska and Kubes, 2013). Early in the wound response, neutrophils are recruited to remove necrotic cells and pathogens through phagocytosis, as well as through release of reactive oxygen species (ROS) and antimicrobial peptides (Kovtun et al., 2018). This first wave of the immune response is subsequently cleared from the wound site by various mechanisms, including apoptosis or clearance by other immune cells such as macrophages (Buckley et al., 2013). The key role of macrophages, however, lies in their ability to act in either a pro- or anti-inflammatory fashion as needed (Kim and Nair, 2019). In the first instance, macrophages that are either already present in the tissue or brought in through the recruitment and differentiation of circulating monocytes assume a pro-inflammatory phenotype (functionally replacing the neutrophils) characterized by the secretion of ROS as well as cytokines, such as IL-1, IL-6 or TNF (Wilkinson and Hardman, 2020). Successful wound healing requires macrophages to switch over to an anti-inflammatory phenotype at later stages in order to promote tissue repair. This can be accomplished either by recruitment of additional monocytes or by a phenotypic change in the macrophages already present (Kim and Nair, 2019). The anti-inflammatory macrophage phenotype is characterized by the secretion of cytokines, such as IL-4, IL-10 and IL-13, along with a host of growth factors, to stimulate epithelial repair. The final stage of the wound inflammatory response is the removal of remaining inflammatory cells, carried out by T cells recruited from the circulation, which clears the way for later stages of wound healing enacted by the epithelium itself (Nosbaum et al., 2016; Wilkinson and Hardman, 2020).

This Review aims to provide a comprehensive overview of the recently discovered roles of inflammation in the injury response process within the context of the respiratory system. Exploring the molecular and cellular mechanisms through which inflammatory signals instruct stem cell activation and differentiation, and how stem cells modulate their surrounding immune landscape, sheds light on the pivotal role of epithelial cells in coordinating the inflammatory response. By elucidating how aberrant inflammation disrupts the repair process and leads to pathological dysregulation, we offer insights into potential therapeutic interventions that target inflammation-mediated tissue regeneration and disease progression.

## Stem cells and immune responses in the respiratory tract

**Fig. 1.**
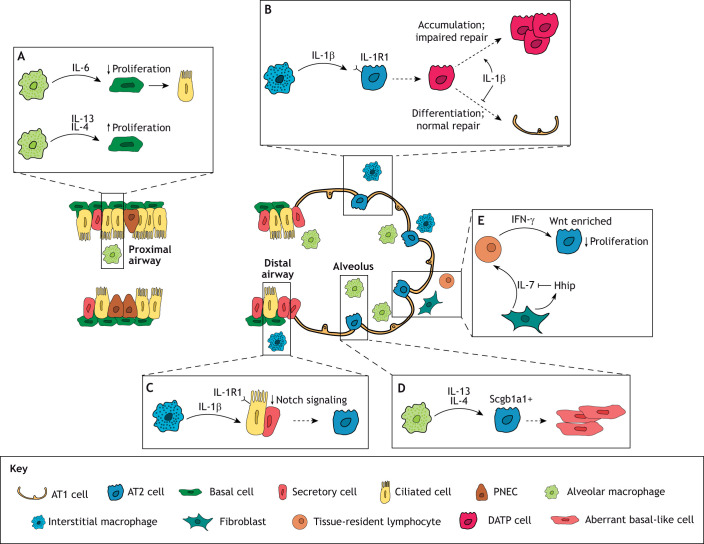
**Impact of immune cell inflammatory responses on stem cell behavior in the lung.** (A) Basal cells of the proximal airway exhibit a range of responses to cytokines secreted by macrophages. Upon exposure to pro-inflammatory IL-6, basal cells suppress their proliferative capacity and differentiate into ciliated cells (Tadokoro et al., 2014). Conversely, exposure to IL-4 and IL-13, which play a major part in regenerative type 2 immune responses, increases the rate of basal cell proliferation in order to maintain an intact tissue barrier (Engler et al., 2020). (B) Following injury to the alveoli of the distal lung, AT2 cells, the facultative stem cells of the alveoli, respond to IL-1β secreted by local interstitial macrophages by transitioning into a DATP state. Upon withdrawal of IL-1β, DATPs differentiate into gas-exchanging AT1 cells to repair the damage. Chronic exposure to IL-1β, however, leads to aberrant accumulation of DATP cells and impaired lung regeneration, potentially leading to diseases such as pulmonary fibrosis and lung cancer (Choi et al., 2020; Hill et al., 2023). (C) Secretory cells of the distal airway also form part of the regenerative response to airway damage. Macrophage-secreted IL-1β suppresses the expression of Notch ligands in ciliated cells, impacting Notch signaling in neighboring secretory cells, which causes them to lose secretory identity and become AT2 cells (Choi et al., 2021). (D) Upon persistent exposure to IL-13, AT2 cells convert into aberrant basal-like cells, one of the key features of pulmonary fibrosis. A subset of AT2 cells expressing low levels of secretory cell marker Scgb1a1 are particularly sensitive to this stimulus (Glisinski et al., 2021). (E) A subset of AT2 cells enriched in Wnt signaling target genes has been shown to have diminished proliferative capacity upon exposure to the cytokine IFNγ, potentially secreted by tissue-resident lymphocytes. Tissue-resident lymphocytes might be stimulated by IL-7 secreted by a subset of lung fibroblasts exhibiting activated Hedgehog signaling, which in turn can be self-regulated by a negative feedback loop involving Hhip (Wang et al., 2023). Solid arrows indicate secretion or regulation of factors; dashed arrows indicate cell responses (e.g. differentiation, proliferation).

Epithelial barrier tissues, including the respiratory tract, are continuously exposed to the external environment and encounter various particles, toxins and microbes. The resulting damage requires an effective response mechanism to rapidly restore tissue integrity and function. The cellular composition and function of the lung epithelium vary along the pulmonary axis from the proximal to the distal lung. The respiratory epithelium is maintained by specific stem and progenitor cells that are intricately governed by their dynamic local environments (Fig. 1). In the proximal airway, the epithelium is primarily maintained by basal cells, which self-renew and differentiate into ciliated and secretory cells (Hong et al., 2004; Rock et al., 2009; Watson et al., 2015). Secretory cells marked by the expression of *Scgb1a1* (secretoglobin 1a1; also known as *CC10* or *CCSP*) and pulmonary neuroendocrine cells (PNECs) also display stem-like features and are capable of reprograming into alternative cell fates as part of tissue repair processes (Kathiriya et al., 2020; Ouadah et al., 2019; Rawlins et al., 2009; Song et al., 2012; Tata et al., 2013). In the distal lung, the role of tissue maintenance and regeneration primarily falls to alveolar type II (AT2) cells, which preserve the gas-exchanging alveolar type I (AT1) cells. Secretory cells are also capable of maintaining the distal airway epithelium and contributing to alveolar lineages when needed (Barkauskas et al., 2013; Choi et al., 2021; Desai et al., 2014; Liu et al., 2019; Rock et al., 2011).

Atypical roles of the immune system have been demonstrated in orchestrating stem cell activity to facilitate efficient tissue restoration after injury. Under normal homeostatic conditions, the activity of innate, tissue-resident immune cells, such as alveolar macrophages in the lung, is suppressed through signaling interactions with the lung epithelium. This suppression occurs via multiple mechanisms, including the anti-inflammatory cytokine interleukin-10 (IL-10), transforming growth factor β (TGF-β), and the CD200–CD200R signaling axis, which involves the activation of the immunoregulatory receptor CD200R by the CD200 ligand prominently expressed in lung epithelial cells. These regulatory mechanisms collectively prevent the triggering of inflammatory responses in the absence of infection or injury (Fernandez et al., 2004; Grunwell et al., 2018; Lemaire and Ouellet, 1996; Raychaudhuri et al., 2000; Shafiei-Jahani et al., 2021; Snelgrove et al., 2008). However, following the disruption of the tissue barrier caused by injury, innate immune cells release pro-inflammatory cytokines, chemokines and signaling molecules to initiate and mediate local inflammatory responses, fostering swift injury repair and regeneration. Resident alveolar macrophages are among the first innate immune cells to respond to damage, alongside infiltrating neutrophils in the lung. These two cell types express high levels of matrix metalloproteinase (MMP)-2 and MMP-9 to remodel the local extracellular matrix (ECM) around the wound, clearing the way for a stronger, more coordinated inflammatory response (Davey et al., 2011; McKeown et al., 2009; Soccal et al., 2000). The recruitment of additional macrophages, along with eosinophils, neutrophils and leukocytes, results in a highly altered immune environment rich in cytokines such as IL-1α, IL-1β, IL-4, IL-13, tumor necrosis factor (TNF; also known as TNF-α) and TGF-β. Some of these cytokines assist the healing process by directly influencing the activity of local stem cells and surrounding stromal cells (Kang et al., 2007; Lappalainen et al., 2012; Mazzon and Cuzzocrea, 2007; Rabolli et al., 2014; Vaz de Paula et al., 2020). As the inflammatory phase progresses, changes in the local microenvironment, including the accumulation of apoptotic neutrophils and anti-inflammatory cytokines, trigger a shift towards anti-inflammatory macrophages. This transition dampens the inflammatory response and supports tissue repair (Mosser and Edwards, 2008). However, dysregulation of this finely balanced immune response can lead to aberrant tissue repair, including excessive accumulation of collagen-producing myofibroblasts, resulting in ECM deposition and fibrotic lesions (Sorokin, 2010; Sutherland et al., 2023). This acute inflammatory response is a common starting point of the damage response of the lung, irrespective of whether the damage is caused by pathogens, chemical or radiation exposure, or inhalation of harmful substances.

## Inflammation and stem cell dynamics in tissue regeneration

The concept of the wound inflammatory response (Box 1) was first proposed by Elie Metchnikoff in the early 20th century (Eming et al., 2017). Since then, damage-induced inflammation has been found to coordinate stem cell activity and fate behaviors during tissue regeneration.

In the proximal airway, the pro-inflammatory cytokine IL-6 limits the proliferation of basal cells and drives their differentiation into ciliated cells (Fig. 1A) (Tadokoro et al., 2014). Conversely, type 2 immune responses – an adaptive immune response commonly associated with allergic inflammation or helminth infection – support barrier maintenance and enhance tissue regeneration in basal cells. Following injury induced by polidocanol treatment, submucosal macrophages with anti-inflammatory M2-like signatures significantly increase, whereas intraepithelial airway macrophages (IAMs) decrease (Engler et al., 2020). Differentiation of CC-chemokine receptor 2 (CCR2)-positive monocytes into IAMs post-injury promotes basal cell proliferation, mediating epithelial regeneration (Fig. 1A).

In the distal lung, myeloid cells are also crucial for alveolar epithelium regeneration. CCR2^+^ monocytes, recruited by AT2 cells expressing CC-chemokine ligand 2 (CCL2) following lung injury, are essential for repair. Moreover, macrophages expressing arginase 1, which is upregulated by IL-4 receptor (IL-4R) signaling triggered by IL-13, regulate AT2 cell behavior to compensate for epithelial loss (Lechner et al., 2017). Interstitial macrophages originating from circulating monocytes also contribute to alveolar regeneration by secreting the pro-inflammatory cytokine IL-1β, which selectively reprograms a subset of AT2 cells expressing IL-1R1, a functional receptor for IL-1β. This reprograming transitions AT2 cells into intermediate cell states, referred to as damage-associated transient progenitors (DATPs), a pre-AT1 transitional cell state (PATS) or alveolar differentiation intermediates (ADIs), ultimately resulting in their differentiation into mature AT1 cells (Fig. 1B) (Choi et al., 2020; Kobayashi et al., 2020; Strunz et al., 2020). Deletion of IL-1R1 on AT2 cells impairs the differentiation of AT2 cells into DATPs, resulting in a failure to replenish AT1 cells and affecting alveolar regeneration (Choi et al., 2020). This highlights the pivotal role of inflammatory signals in modulating the regenerative potential of AT2 cells and initiating their differentiation during tissue regeneration. IL-1 signaling also drives conversion of distal airway secretory cells into AT2 cells, facilitating alveolar regeneration (Choi et al., 2021). IL-1 inhibits the expression of Notch ligands in ciliated cells expressing IL-1R1, reducing Notch activity in secretory cells (Fig. 1C). This molecular cascade turns off the genetic programs required for secretory cell identity and activates those for AT2 cells, leading to secretory cell differentiation, demonstrating the role of inflammation in modulating niches to govern stem cell behaviors.

The successful resolution of these injury-related responses depends on the transient nature of inflammatory signals. Prolonged exposure to cytokines can lead to disease development, although what causes the precise transition from regenerative to pathological inflammation remains elusive. Persistent IL-1β signaling results in the accumulation of intermediate cell states and impedes AT1 cell maturation (Fig. 1B), both features observed in chronic lung diseases such as pulmonary fibrosis and lung cancer (Choi et al., 2020; Hill et al., 2023). Likewise, sustained IL-13 exposure prompts the conversion of AT2 cells into aberrant basal-like cells, a hallmark of pulmonary fibrosis, with a subset of AT2 cells that express low levels of the secretory cell marker Scgb1a1 alongside the canonical AT2 marker Sftpc showing particularly strong responses to this stimulus (Fig. 1D) (Glisinski et al., 2021). Intriguingly, another subset of AT2 cells enriched in Wnt signaling target genes, which represent a distinct subpopulation of alveolar epithelial progenitors with potent stem cell activity, loses proliferative capacity in the presence of interferon γ (IFNγ), unlike the remainder of the AT2 cell pool (Fig. 1E) (Wang et al., 2023). This response was initially described in the context of maladaptive responses of tissue-resident lymphocytes to viral infections. In this context, specialized fibroblasts can stimulate lymphocyte secretion of IFNγ by secreting IL-7; IL-7 can in turn be self-regulated through modulation of the Hedgehog signaling pathway (particularly through the signaling antagonist Hhip). The broad range of immune cell types capable of secreting IFNγ, however, suggests that it is an important factor in the immune-based regulation of stem cell function. These findings represent a significant step toward understanding how impaired regeneration can lead to the onset of pathological traits. Given the dual role of inflammatory signals in initiating regeneration and fostering pathological responses, the intricate spatiotemporal regulation of these processes presents an attractive target for mitigating aberrant inflammatory and fibrotic responses.

## Stem cells in inflammatory niche remodeling

The interplay between the epithelium, its stem cells and inflammatory responses is bidirectional and complex. The airway epithelium, constituting the mucosal barrier, expresses various pattern recognition receptors (PRRs) that trigger innate immune responses against pathogens through IFNs and their downstream signaling. Moreover, airway epithelial cells produce and secrete various cytokines and chemokines that modulate immune cells. In the context of asthma and other inflammatory conditions, airway epithelial cells release factors such as CCL20, thymic stromal lymphopoietin (TLSP; also known as KLK11), IL-33, IL-25 and granulocyte-macrophage colony-stimulating factor (GM-CSF; also known as CSF2). These mediators orchestrate complex interactions between the epithelium and various immune cells. For instance, upon infection, epithelial-derived IL-33 activates invariant natural killer T (iNKT) cells via IL-33R, leading to IL-13 production, which modulates the activation of macrophages and monocytes (Byers et al., 2013; Kim et al., 2008). TLSP released from airway epithelial cells in response to allergic stimuli contributes to asthmatic responses (Gauvreau et al., 2014). Additionally, epithelial-derived IFNβ induces dendritic cells (DCs) to upregulate CCL28, enhancing the recruitment of CCR10^+^ T helper 2 (T_H_2) cells to the lungs (Cheung et al., 2010; Grayson et al., 2007).

**Fig. 2.**
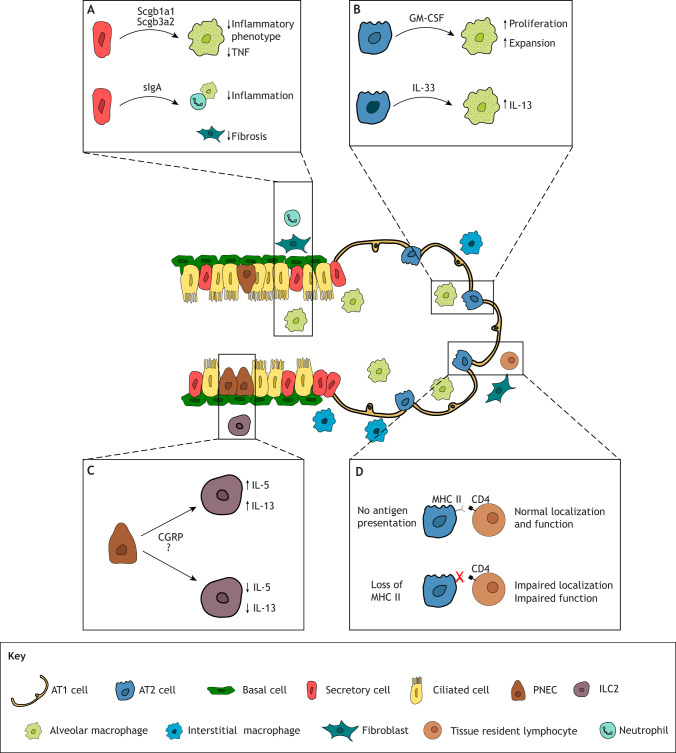
**Modulation of immune function by different stem cell compartments of the airway.** (A) Secretory cells regulate immune responses in the airway by suppressing inflammatory responses in macrophages through the secretion of Scgb1a1 and Scgb3a2, and in other inflammatory cells, such as neutrophils, through the secretion of IgA. The absence of secreted IgA (sIgA) results in increased inflammation and fibrosis upon bacterial infection (Polosukhin et al., 2012, 2017). (B) AT2 cells can both sustain and modulate local alveolar macrophage populations. Continuous secretion of GM-CSF by AT2 cells is required to maintain proliferation and expansion of the alveolar macrophage pool; loss of GM-CSF signaling impairs regeneration and leads to lung fibrosis (Gschwend et al., 2021; Moore et al., 2000). AT2 cells can also secrete IL-33 to stimulate IL-13 production in alveolar macrophages, which in turn impacts basal cell behavior (Byers et al., 2013). (C) PNECs of the airway can modulate ILC2 cells in their local environment by secreting CGRP, which affects ILC2 secretion of cytokines, such as IL-5 and IL-13. However, as CGRP has been shown to both enhance and inhibit interleukin secretion in ILC2s, the precise mechanism and any additional factors involved remain unclear (Nagashima et al., 2019; Sui et al., 2018; Wallrapp et al., 2019). (D) AT2 cells express high levels of MHC II, a molecule typically utilized by the antigen-presenting cells of the immune system to drive adaptive immune responses through interactions with the CD4 receptor found on helper T lymphocytes. Although AT2 cells do not use MHC II to present antigens, removing MHC II from these cells distorts the local immune niche by affecting the localization and function of tissue-resident memory T cells (Hasegawa et al., 2017; Shenoy et al., 2021; Toulmin et al., 2021).

Stem cells play a central role in driving early inflammatory responses by producing immunomodulatory molecules that facilitate tissue regeneration after injury. Recent studies have highlighted the importance of stem cells, particularly AT2 cells, in regulating both local innate and adaptive immune responses for tissue homeostasis and regeneration. AT2 cells secrete GM-CSF, which regulates the proliferation and expansion of local alveolar macrophages, a relationship established during prenatal development that remains crucial for lung homeostasis throughout life (Fig. 2B) (Gschwend et al., 2021). The significance of this tissue-immune interaction in lung regeneration is further underscored by findings that loss of GM-CSF leads to aberrant regeneration and increased severity of fibrosis in bleomycin-induced lung injury (Moore et al., 2000). Additionally, a subset of AT2 cells can stimulate macrophages to produce IL-13 via IL-33 secretion (Fig. 2B) (Byers et al., 2013). As previously mentioned, macrophage-derived IL-13 itself can influence basal cell behavior during tissue injury. These findings demonstrate the complex interplay between stem cell and immune responses in the regeneration process. Given the stem cell properties of AT2 cells, the regeneration process can become dysregulated if these cells continuously produce IL-33, potentially distorting both airway immune function and tissue repair. Evidence of such dysregulation has been observed in chronic obstructive pulmonary disease (COPD), where airway tissue from patients displays increased IL-33 expression (Byers et al., 2013). AT2 cells also express high levels of the type II major histocompatibility complex (MHC II), a molecule typically found on antigen-presenting immune cells that governs adaptive immune responses through direct interactions with CD4^+^ T cells (Hasegawa et al., 2017; Shenoy et al., 2021; Toulmin et al., 2021). Although AT2 cells do not fully utilize MHC II for bona fide antigen presentation, ablation of MHC II alters the localization and function of tissue-resident T cells, distorting the local immune niche and leading to poorer outcomes following viral challenge (Fig. 2D) (Shenoy et al., 2021; Toulmin et al., 2021).

Additional types of respiratory stem cells possess the ability to modulate their local immune niche. PNECs influence the responses of local type 2 innate lymphoid cells (ILC2s), which can produce type 2 cytokines and mediate type 2 immune response, through their characteristic secretion of calcitonin gene-related peptide (CGRP) (Sui et al., 2018). However, the precise nature of this interaction is still not fully understood, as CGRP has been shown to both stimulate and inhibit the ILC2 response. This response involves the secretion of cytokines such as IL-5 and IL-13 and the subsequent recruitment of additional inflammatory cells such as eosinophils (Fig. 2C) (Nagashima et al., 2019; Sui et al., 2018; Wallrapp et al., 2019). These opposing findings suggest that additional, as-yet-unknown, factors govern the remodeling of the local immune niche by neuroendocrine cells. Secretory cells primarily function to suppress inflammatory responses and maintain immune homeostasis through the secretion of characteristic proteins, such as Scgb1a1 and Scgb3a2 (Fig. 2A) (Laucho-Contreras et al., 2015; Yoneda et al., 2016). In addition, secretory cells of the lung and other mucosal tissues regulate immune homeostasis via secretory IgA, the absence of which leads to bacterial infection-induced fibrosis (Fig. 2A) (Polosukhin et al., 2012, 2017).

Overall, stem cells throughout the respiratory tract are capable of remodeling their local immune niches, driving a broad range of responses to damage and infection. Just as the regeneration process is tightly regulated, the interactions between stem cells and the immune system must also be carefully controlled. Aberrant immune phenotypes and regenerative responses have the potential to create feedback loops, distorting both processes and leading to pathological outcomes.

## Inflammation and epithelial cell memory in tissue maintenance and regeneration

**Fig. 3.**
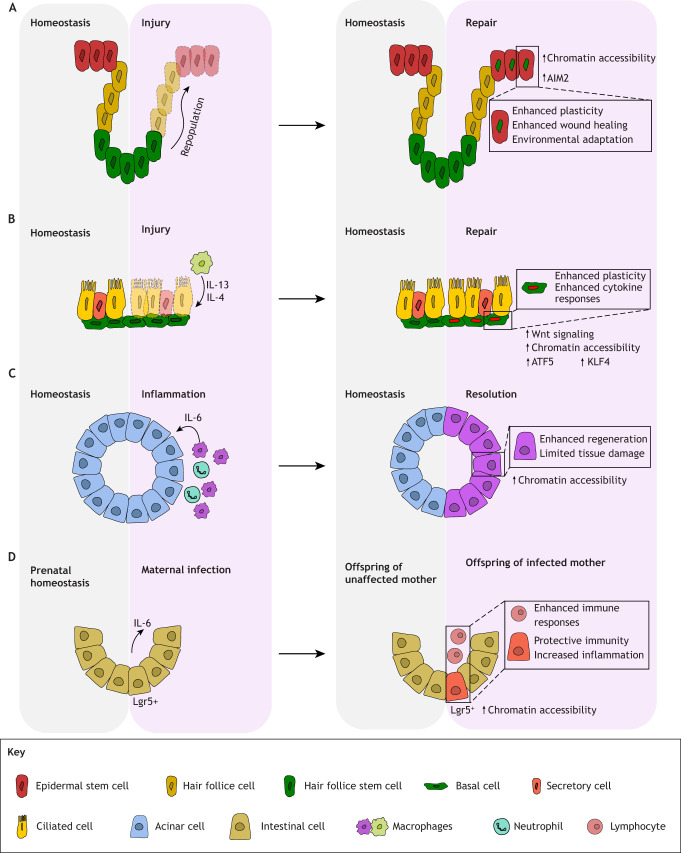
**Inflammatory memory confers altered stem cell characteristics and functionality across numerous tissues.** (A) Following wounding of the epidermis (left), hair follicle stem cells repopulate the wounded area (right). The new hair follicle-derived epidermal stem cells carry epigenetic memory of their origins and exhibit enhanced plasticity and healing capacity, making them more effective at responding to repeated wounding (Naik et al., 2017; Gonzales et al., 2021). (B) Following airway injury and subsequent exposure of basal cells to macrophage-derived IL-4 and IL-13 (left), airway basal cells repair the damage (right) and retain memory of their exposure to the reparative stimuli, as evidenced by increased chromatin accessibility, persistent Wnt signaling and upregulation of transcription factors ATF3 and KLF5 (Ordovas-Montanes et al., 2018). (C) Upon exposure to transient inflammatory signals such as IL-6 (left), pancreatic acinar cells gain inflammatory memory evidenced by increased chromatin accessibility and enhanced regenerative capabilities, subsequently leading to decreased tissue damage upon repeated insults (right) (Del Poggetto et al., 2021). (D) Inflammatory memory can even be passed between mother and progeny. Maternal Lgr5^+^ intestinal stem cells are exposed to IL-6 following infection (left); subsequently, offspring display epigenetic remodeling in their intestinal stem cells despite not being directly exposed to the infection (right). Lgr5^+^ stem cells in the offspring have increased chromatin accessibility, leading to enhanced immune and inflammatory responses, which provide increased protection from subsequent infections (Lim et al., 2021).

Our body possesses an extraordinary ability to remember previous encounters with pathogens and insults, quickly mounting effective responses upon re-exposure. This memory response, a hallmark of adaptive immunity, has also been recognized in the innate immune system, where it is termed ‘trained immunity’, elucidating the concept of inflammatory memory. Recent studies have revealed that epithelial stem cells exhibit remarkable adaptive cellular reprograming in response to repeated inflammatory clues, expanding the scope of memory mechanisms beyond immune cells. A study on wound healing in the epidermis showed that prior inflammatory exposure endows stem cells in the hair follicle bulge with enhanced and accelerated repair properties, providing evidence of inflammatory memory in epithelial cells (Fig. 3A) (Naik et al., 2017). Stem cells can retain diverse epigenetic changes specific to different stimuli, such as wound healing or changes in cell fate driven by tissue repair needs, facilitating effective responses to repeated insults (Gonzales et al., 2021; Naik et al., 2017).

The pulmonary epithelium also exhibits a form of immune-related memory, particularly evident in chronic inflammatory conditions. In chronic rhinosinusitis, a persistent inflammation of the upper airways and mucosa of the paranasal sinuses, airway basal cells retain markers of their exposure to IL-4 and IL-13. These markers include sustained Wnt signaling and upregulation of transcription factors ATF3 and KLF5, associated with maintaining an undifferentiated state, chromatin opening and oncogenesis (Fig. 3B) (Ordovas-Montanes et al., 2018). This inflammatory memory enhances basal cell plasticity and increases cell responsiveness to subsequent cytokine challenges. Given the similarities between epidermal and respiratory tract barrier functions and the constant stem cell–immune cell crosstalk in both tissues, various stem cell populations in the lung likely bear a range of epigenetic marks from past exposures. However, the extent and specificity of these ‘memories’ in various lung stem cell types remain to be fully elucidated.

Inflammatory memory has been observed in various tissues, demonstrating its broad relevance in biology. In the pancreas, acinar cells exposed to transient inflammation were found to display enhanced regenerative activity and limited tissue damage upon subsequent challenges (Fig. 3C) (Del Poggetto et al., 2021). Intriguingly, inflammatory memory can also be transmitted across generations. A study by Lim et al. revealed that maternal infection during pregnancy in mice can confer long-lasting epigenetic memory to offspring through a mechanism involving IL-6. Specifically, maternal exposure to IL-6, either through direct administration or as a result of infection, led to epigenetic remodeling in the intestinal stem cells of adult offspring. This remodeling was characterized by changes in chromatin accessibility and transcriptional profiles, resulting in an enhanced regenerative capacity in response to subsequent infections or tissue damage (Fig. 3D) (Lim et al., 2021). These findings raise important questions about the long-term consequences of inflammatory memory in stem cell populations. As stem cells age, they might accumulate various epigenetic marks associated with inflammatory memory. Although these marks can confer protective effects, they might also contribute to the development of senescence and the production of pro-inflammatory factors related to ‘inflammaging’, discussed in more detail below. This raises the question of whether inflammatory memory should always be maintained or whether resetting the epigenetic landscape might sometimes be advantageous for tissue homeostasis and regeneration.

## Inflammation and stem cell aging in tissue maintenance and regeneration

Significant progress has been made in understanding the aging program in stem cells over the past decades. Increasing evidence points to defects in the maintenance of homeostasis and a decline in physiological function in tissues, coinciding with the aging process and the onset of senescence in stem and progenitor cells. However, despite this progress, the underlying mechanisms remain relatively enigmatic due to the complexity of regulatory programs, the heterogeneity of senescent populations and the lack of definitive markers for senescent cells. Cellular senescence is characterized by distinct hallmarks, including permanent cell cycle exit, which can directly impact stem cell activity, and the release of specific substances collectively known as the senescence-associated secretory phenotype (SASP). Given that many of the components of SASP, such as IL-6 and IL-8, are inflammatory mediators, senescent cells can actively modulate the surrounding niche. Depending on the composition, localization and duration (e.g. acute or chronic) of SASP, senescent cells can exert either beneficial or detrimental effects on tissue function and regenerative responses (Di Micco et al., 2021).

**Fig. 4.**
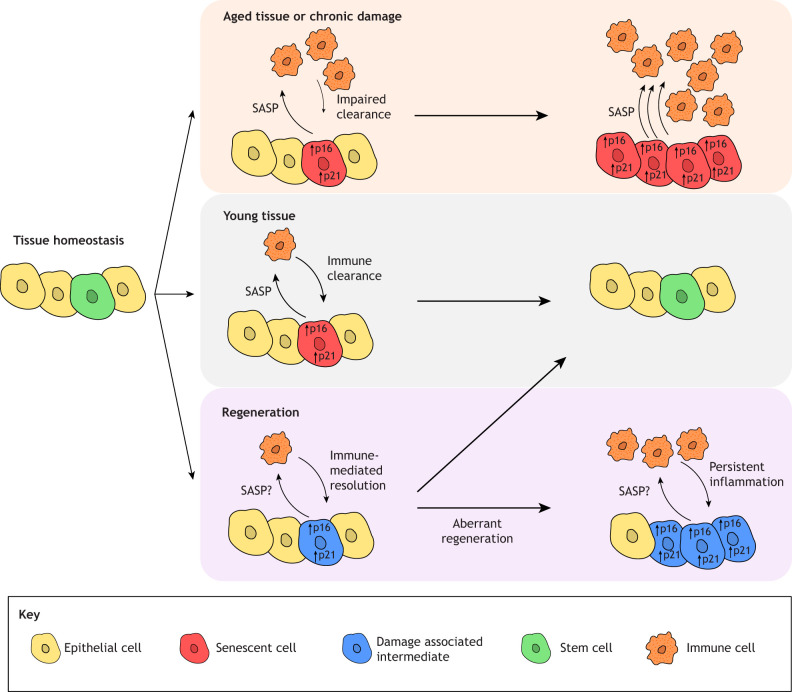
**The multifaceted roles of senescence in inflammation and damage response.** A broad range of factors can drive cells, including stem cells, into a state of senescence, characterized by permanent cell cycle exit and secretion of a broad range of cytokines known as the senescence-associated secretory phenotype (SASP), which allows senescent cells to modulate their local immune environment. Senescent cells can be cleared by the immune system in order to restore tissue homeostasis (middle). The composition of the SASP and the duration of secretion dictates whether senescent cells act in a beneficial or detrimental fashion (Di Micco et al., 2021). Aging, as well as chronic or repeated damage, both impact this process by driving inflammaging, a low level of chronic inflammation coupled with impaired immune-driven clearance of senescent cells (top). Accumulation of senescent cells and an increased inflammatory environment result in an overall decrease in organ function that is exacerbated with age. The process of normal senescent cell repair is mirrored by the regenerative response of AT2 cells (bottom). Transient damage-associated intermediate cells display many hallmarks of senescent cells, including expression of cell cycle regulators p16 and p21 and secretion of numerous SASP factors. Aberrant regeneration follows a similar pattern to that of aged or chronically damaged tissue, with an accumulation of intermediate cell states that continuously secrete inflammatory factors and evade clearance by the local immune system (Choi et al., 2020; Kobayashi et al., 2020; Strunz et al., 2020).

Inflammation is a key regulator in the initiation and maintenance of the senescent cell state. During aging and repeated injury, organs throughout the body exhibit ‘inflammaging’, a low level of chronic inflammation marked by an increase in pro-inflammatory factors (Chen et al., 2018; Ferrucci and Fabbri, 2018; Franceschi et al., 2018). These factors reprogram stem cells and their niches to acquire senescent characteristics, altering regenerative capacity and tissue repair processes (Fig. 4). The decrease in the number and activity of stem cells, coupled with aging and chronic inflammation, is a conserved feature across diverse tissues, including the lung (Chen et al., 2021; Funk et al., 2023). As outlined in previous sections, during injury repair, IL-1β signaling drives AT2 cells into a DATP state, which bears many hallmarks of senescent cells, such as increased expression of cell cycle regulators p16 and p21 (also known as CDKN2A and CDKN1A, respectively), several SASP proteins including amphiregulin (Areg) and activation of nuclear factor (NF)-κB signaling (Choi et al., 2020; Kobayashi et al., 2020; Strunz et al., 2020). Given the transient nature of these intermediate cells, it is unclear whether DATPs are truly senescent or simply co-opting part of the senescence program to make necessary modifications to the local environment during regeneration. However, persistence of this state is closely linked to the development of chronic lung diseases (Fig. 4) (Adams et al., 2020; Habermann et al., 2020; Han et al., 2024; Liu et al., 2024). Furthermore, cells displaying senescent characteristics can also serve as intermediaries between the inflammatory response and stem cells required for tissue repair. A population of adventitial fibroblasts, located in the outermost layer of connective tissue of vessels in the airway, exhibiting senescent features induced by IL-1 signals from interstitial macrophages, enhanced the regenerative capabilities of airway secretory cells through epiregulin (Ereg) secretion (Reyes et al., 2022). Additionally, a recent study in aged lungs in mice has shown that aging increases the expression of injury-induced senescence genes, including those related to inflammation and SASP, in AT2 cells (Liang et al., 2023). These findings suggest that the regenerative regulation induced by tissue injury and the senescence process in stem cells share similar inflammatory programs. Determining the extent of overlap between these mechanisms is crucial for understanding both injury repair and aging.

The overall quality of immune responses to any challenge diminishes with age, with cells across the entire immune system exhibiting impaired functionality. This process forms a feedback loop to promote epithelial transitional states associated with senescent gene signatures. As the immune system becomes gradually less capable of supporting the regenerative potential of stem cells and restoring the original tissue architecture, the local immune landscape is further distorted. Consequently, improving tissue maintenance with age will likely require addressing not only the functionality of tissue-resident stem cells but also the immune system as a whole.

## Conclusions and perspectives

Collectively, the studies highlighted in this review demonstrate that inflammation coordinates the properties of epithelial stem cells to direct finely tuned responses that mitigate tissue damage, repair wounds and restore tissue homeostasis and function. Inflammatory signals not only instruct stem cell activity in the short term but also imprint long-lasting memory onto their epigenetic architecture, enabling faster responses upon subsequent damage. This program is closely linked to the senescence of stem cells during tissue aging, particularly under conditions of low-grade chronic inflammation or inflammaging. Notably, epithelial stem cells are capable of both sensing stimuli and changes from the external environment, as well as instructing the immune system to initiate and regulate inflammatory responses, harnessing both its innate and adaptive elements.

Despite these advances, several key areas of knowledge are currently lacking and warrant further investigation. In the context of tissue regeneration, immune regulation is complex and coordinated by multifaceted interactions constituting niches. Future studies should further address which cell types are involved in this interplay and how signaling pathways interconnect to ensure regenerative immunity. Increasing evidence is uncovering the instructive impact of epithelial stem cells in reprogramming fibroblast states into inflammatory or fibrotic niches, which in turn aligns immune responses. However, the precise mechanisms of this reprogramming and its effects on long-term tissue homeostasis remain unclear. Therefore, mapping sequential cellular interactions and identifying key regulatory networks among epithelial, stromal and immune cells will enhance our understanding of immunological spatial niches in maintaining tissue homeostasis and regeneration. The molecular switches determining whether inflammation promotes regeneration or pathological responses are yet to be fully understood. How these cellular and molecular programs are altered and associated with diverse chronic lung diseases and aging remains to be elucidated and warrant further investigation. Revealing the complex mechanisms underlying the dynamic dialogue between niches governing immune responses – conserved across various tissue contexts – is crucial for understanding how tissues maintain and restore homeostasis during wound repair. Comprehending this program will help unlock the mechanisms behind chronic diseases, including those associated with tissue aging.
